# Comparative genomics of Mollicutes-related endobacteria supports a late invasion into Mucoromycota fungi

**DOI:** 10.1038/s42003-023-05299-8

**Published:** 2023-09-18

**Authors:** Reid Longley, Aaron Robinson, Julian A. Liber, Abigail E. Bryson, Demosthenes P. Morales, Kurt LaButti, Robert Riley, Stephen J. Mondo, Alan Kuo, Yuko Yoshinaga, Chris Daum, Kerrie Barry, Igor V. Grigoriev, Alessandro Desirò, Patrick S. G. Chain, Gregory Bonito

**Affiliations:** 1 Los Alamos National Laboratory, Los Alamos, NM USA; 2 Department of Microbiology and Molecular Genetics, Michigan State University, East Lansing, MI 48824 USA; 3 Department of Biology, Duke University, Durham, NC 27704 USA; 4 Department of Plant Soil and Microbial Sciences, Michigan State University, East Lansing, MI 48824 USA; 5 United States Department of Energy Joint Genome Institute, Lawrence Berkeley National Laboratory, Berkeley, CA 94720 USA; 6 Department of Agricultural Biology, Colorado State University, Fort Collins, CO 80521 USA; 7 Department of Plant and Microbial Biology, University of California Berkeley, Berkeley, CA 94720 USA

**Keywords:** Fungal evolution, Phylogenetics

## Abstract

Diverse members of early-diverging Mucoromycota, including mycorrhizal taxa and soil-associated Mortierellaceae, are known to harbor Mollicutes-related endobacteria (MRE). It has been hypothesized that MRE were acquired by a common ancestor and transmitted vertically. Alternatively, MRE endosymbionts could have invaded after the divergence of Mucoromycota lineages and subsequently spread to new hosts horizontally. To better understand the evolutionary history of MRE symbionts, we generated and analyzed four complete MRE genomes from two Mortierellaceae genera: *Linnemannia* (MRE-L) and *Benniella* (MRE-B). These genomes include the smallest known of fungal endosymbionts and showed signals of a tight relationship with hosts including a reduced functional capacity and genes transferred from fungal hosts to MRE. Phylogenetic reconstruction including nine MRE from mycorrhizal fungi revealed that MRE-B genomes are more closely related to MRE from Glomeromycotina than MRE-L from the same host family. We posit that reductions in genome size, GC content, pseudogene content, and repeat content in MRE-L may reflect a longer-term relationship with their fungal hosts. These data indicate *Linnemannia* and *Benniella* MRE were likely acquired independently after their fungal hosts diverged from a common ancestor. This work expands upon foundational knowledge on minimal genomes and provides insights into the evolution of bacterial endosymbionts.

## Abstract (web summary)

Probing the evolution of bacterial endosymbionts in Mucoromycota - Mollicutes-related endobacteria (MRE) - reveals that the genera Linnemannia and Benniella acquired MRE after host divergence from a common ancestor.

## Introduction

Cellular bacterial endosymbionts are common across the eukaryotic tree of life. Genomes of these endosymbionts are often reduced in size and central metabolism functions leading to a reliance on metabolic crosstalk and exchange with eukaryotic hosts^1–4^. Long-term bacterial endosymbionts frequently harbor uncharacteristic genome attributes such as low numbers of repeats and pseudogenes and a lower GC content^3^. Fungi host intracellular bacterial endosymbionts, known as endobacteria. Particularly, endobacteria are common in diverse lineages of early diverging Mucoromycota fungi. Among fungal endobacteria, lineages related to *Burkholderia*, known as *Burkholderia*-related endobacteria (BRE) are the best studied and are known to impact host functioning, reproduction, and ecology^5–10^. Mucoromycota fungi can also host lineages of endobacteria related to rapidly evolving, specialized Mollicutes cellular endosymbionts^11–15^. This clade, known as Mollicutes-related endobacteria (MRE), had long been visualized within the hyphae of Mucoromycota fungi but were only recently determined to be related to Mollicutes^16,17^. MRE in the Mortierellaceae have been characterized as being of similar size to Mycoplasma cells (300–800 nm) and have been shown to range in size from approximately 310–540 nm^12,18^. MRE can colonize Mortierellaceae hyphae at densities above 10^7^ bacterial cells/mg of fungal tissue^12^. MRE are detected in diverse lineages of Mucoromycota, but their evolutionary history and impact on host physiology remain largely undetermined^12,15,19^. However, it was determined in *Gigaspora margarita* that more carbon is directed from fungal hosts to MRE than BRE, possibly indicating an increased cost of hosting MRE compared to BRE^20^.

The first sequenced MRE genomes were derived from arbuscular mycorrhizal fungi (AMF); these genomes revealed typical endosymbiont features including reduced genome size and metabolic capacity compared to closely related Mollicutes^13,19,21^. Some fungal hosts (e.g., *Rhizophagus irregularis*, *Rhizophagus clarus*) harbor a single MRE population while others (*Diversispora epigaea, Claroideoglomus etunicatum* and members of the Endogonales) harbor multiple^11,13,19,22^. Some MRE from mixed populations are more closely related to MRE from distant hosts than MRE from the same host, raising questions about the origins and acquisition of MRE in Mucoromycota^19^. There are multiple working hypotheses regarding the timing of MRE acquisition by Mucoromycota fungi. The ‘early-invasion hypothesis’ postulates a single origin whereby an MRE ancestor invaded a common ancestor of Mucoromycota, followed by co-diversification alongside fungal hosts and subsequent loss from some hosts^23^. The ‘late-invasion hypothesis’ postulates that a MRE ancestor invaded Mucoromycota after the divergence of the three sub-phyla. These hypotheses do not preclude the possibility of multiple invasions, or horizontal transfer of endosymbionts between fungal hosts^23^. An assessment of these hypotheses is important to understanding the ecological implications of how MRE influence fungal ecology and plant-fungal interactions. Additional sampling of MRE genomes, particularly in the Mortierellaceae, which harbor single populations of the most basal lineages of MRE observed, is needed to interrogate these hypotheses^12^.

To address these hypotheses we present four complete, circular MRE genomes from two Mortierellaceae genera (*Linnemannia* and *Benniella*) and a novel comparative genomics analysis inclusive of previously sequenced MRE genomes. We anticipated that Mortierellaceae MRE genomes would have similar features to those from AMF and Endogonales, including reduced genome size and metabolic capacity, and that the loss of DNA repair genes could account for the unusually rapid rate of evolution within MRE. We expected that cophylogeny among MRE and their fungal hosts would support the ‘early-invasion’ hypothesis, and that a lack of cophylogeny would be evidence for a ‘late-invasion’. This work presents the first complete MRE genome assemblies from Mortierellaceae hosts and expands our knowledge on the evolution of these endobacteria through phylogenetics and comparative genomics.

## Results

### FISH imaging confirms internalization of MRE

**Fig. 1:**
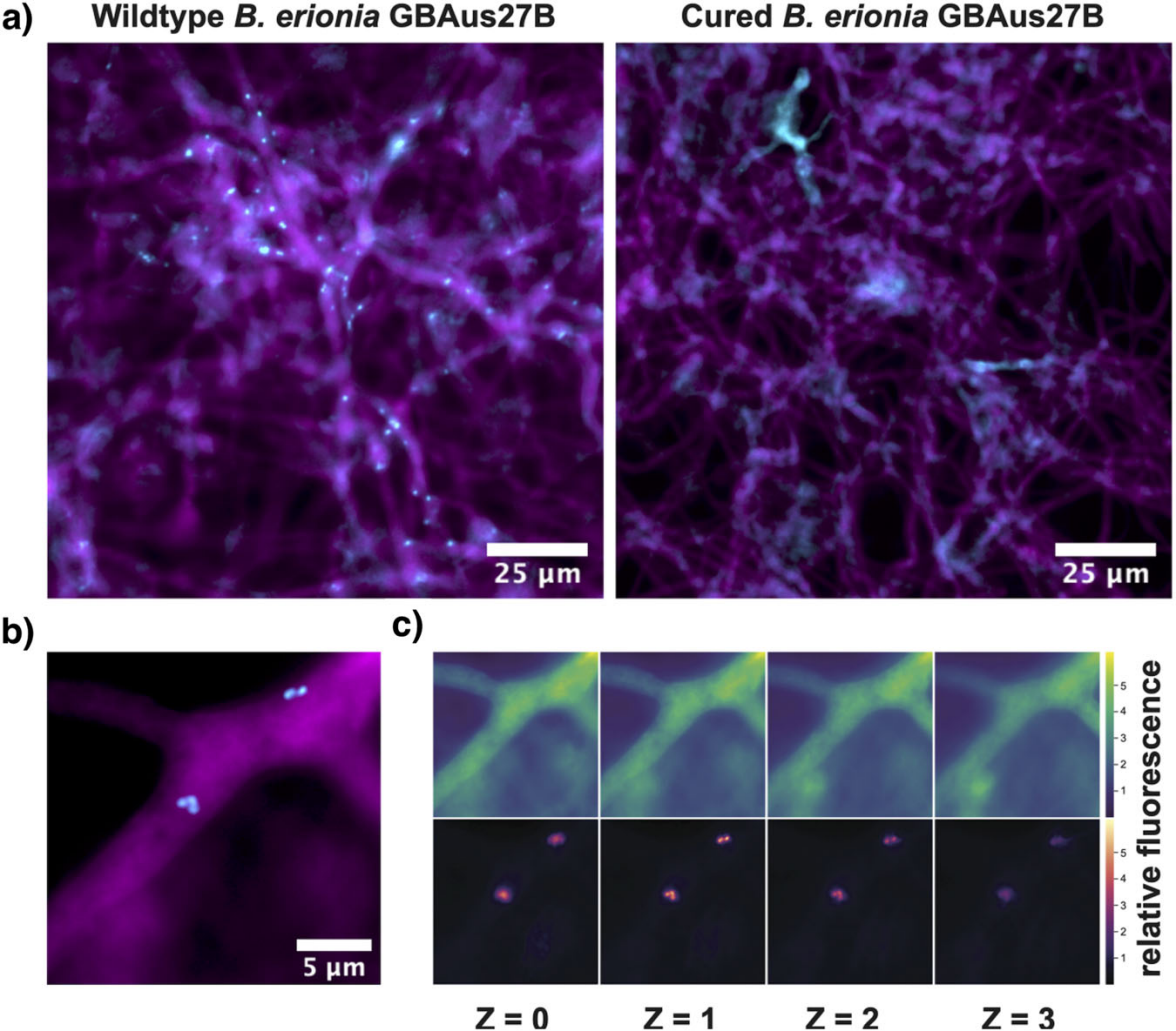
FISH imaging demonstrates intracellular localization of MRE in *Benniella erionia* GBAus27B. **a** FISH imaging in wildtype and cured *Benniella erionia* GBAus27B. MRE and fungal mycelia are visualized using the universal 16S rRNA (Cyan) and 18S rRNA (Magenta) showing specificity of the bacterial probe in only the uncured fungus. Non-specific, amorphous staining was observed in the cured sample. **b** A high magnification image of the signals in (**a**) displays distinct bacterial morphologies. **c** Relative fluorescence intensity across the Z-axis shows that the peak intensities of the bacteria correlate with the 18S rRNA of fungal mycelia indicating that these symbionts are internalized symbionts.

MRE originally detected in isolates of Mortierellaceae as described by ref. ^12^, were confirmed through PCR prior to genome sequencing of the fungal host. Visual comparisons of cured and uncured cultures of *Benniella erionia* GBAus27B with fluorescence microscopy showed that fungal mycelia from the uncured isolate were well colonized by bacteria, as labeled by the 16S rRNA probe (Cyan, Fig. 1a, left panel), while the cured isogenic strain (right panel) displayed non-specific binding that lacked distinguishable bacterial features apparent at higher magnification (Fig. 1b). MRE cells in Fig. 1b ranged in size from approximately 471 to 662 nm. Relative fluorescence across the Z-axis demonstrated that the peak fluorescence of the 16S rRNA probe correlated with the peak intensities in the 18S rRNA probe, verifying that these bacteria were located intracellularly within the mycelia (Fig. 1c). A visual survey of the mycelial network showed bacteria distributed ubiquitously across the fungal colony (Supplemental Movie 1).

### Complete genome assemblies of mortierellaceae hosts and MRE

The four circularized MRE genome assemblies obtained from Mortierellaceae ranged in size from 326,911 bp (*Linnemannia elongata* AD073) to 615,212 bp (*Benniella* sp. AD185) (Supplementary Data 1). CheckM results demonstrated that these genome sequences had low levels of contamination (<2) while completeness estimates ranged from 45.4% to 56.7% based upon a Mollicutes reference genome dataset^24^. The two MRE-L had smaller genome sizes, fewer genes and lower GC content compared to MRE-B (Supplementary Data 1), as well as reduced GC content (22.8%, 24.7%) compared to MRE representatives from the majority of AMF and Endogonales hosts.

Fungal genomes for *Linnemannia elongata* AD073, *Linnemannia gamsii* AM1032 and *Benniella* sp. AD185 were assembled as part of sequencing their respective MRE and the previously published *Benniella erionia* GBAus27B fungal genome was utilized^25^. Genomes were largely complete, with >97.5% of BUSCO genes detected and CDS completeness >93%^26^. Total gene counts ranged between 13,851 (AD073) and 15,422 (AD185) genes (Supplementary Table 1).

### Lack of cophylogeny provides evidence for late invasion hypothesis

**Fig. 2:**
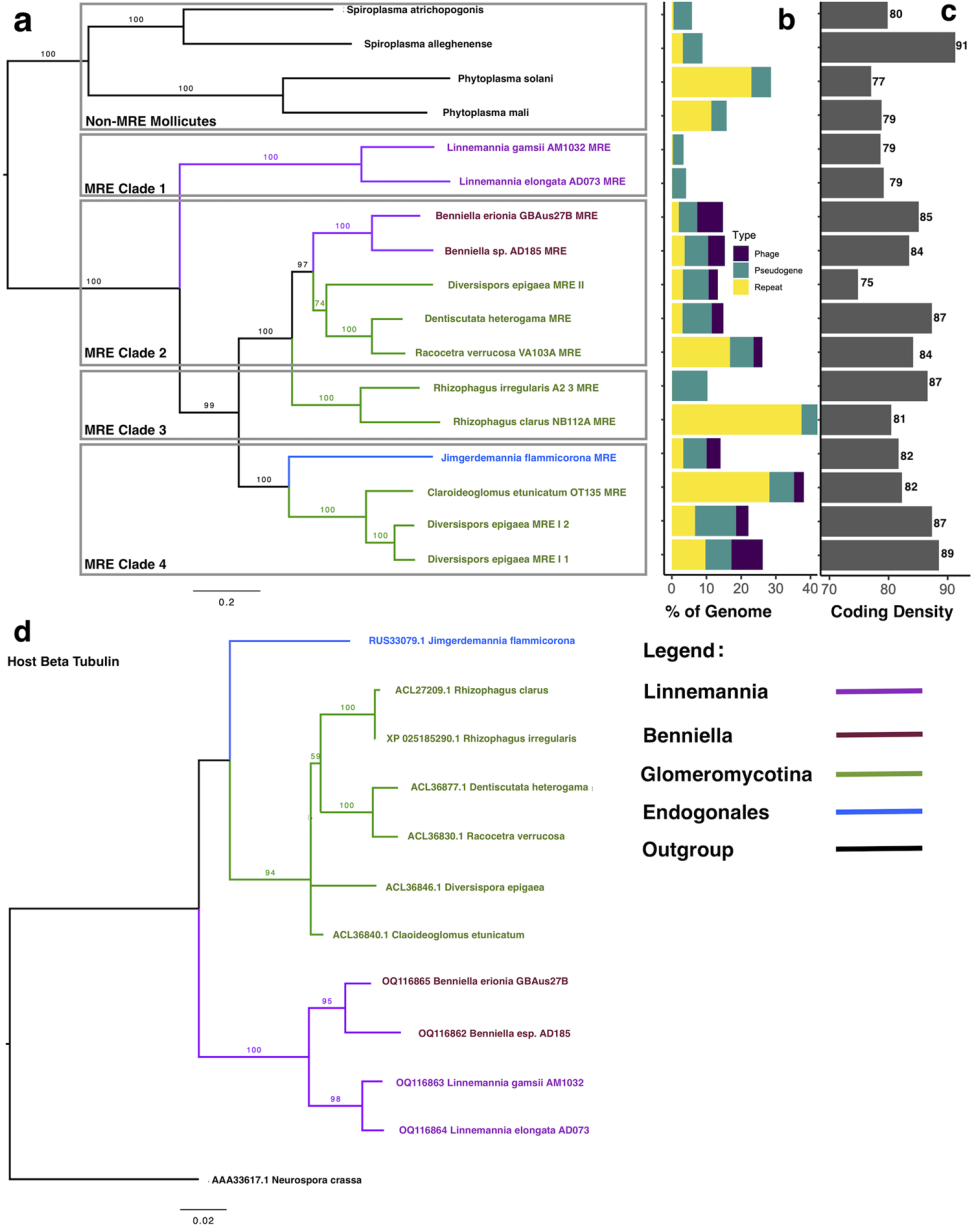
Mortierellaceae MRE differ in phylogenetic placement and genomic content. **a** Maximum likelihood phylogeny of MRE and selected *Phytoplasma* and *Spiroplasma* taxa indicates that MRE form four distinct phylogenetic clades. *Benniella* MRE and *Linnemannia* MRE cluster in separate phylogenetic clades. **b** Percentage of each genome composed of pseudogenes, repeat regions, and phage sequences. **c** Coding density of each genome, (**d**) Host phylogeny created from beta-tubulin genes. Branch labels indicate bootstrap values (100 replicates). Bootstrap values >50 are shown.

Phylogenetic reconstructions based upon ten single copy orthologs showed that MRE clustered into four distinct lineages. The annotations for these single copy orthologs are shown in Supplementary Table 2. The MRE from Mortierellaceae were split into two lineages with MRE-L forming a distinct lineage (clade 1) and MRE-B clustering within clade 2 containing various MRE from AMF (Glomeromycotina). MRE from *Rhizophagus* hosts also formed a distinct lineage (clade 3) and clade 4 was composed of an Endogonales representative and various AMF MRE (Fig. 2a). As previously described, the three MRE representatives of *D. epigaea* were split between two lineages^19^. As expected, the fungal host phylogeny based on beta-tubulin showed high bootstrap support for nodes separating Glomeromycotina and Mortierellaceae (Fig. 2d).

### Genomic features consistent with endobacterial lifestyle

MRE-L genomes had fewer pseudogenes, repeat regions, and phage content compared to other MRE taxa (Fig. 2b, Supplementary Data 1). Pseudogenes and repeat regions accounted for 3.4 and 4.1% of the MRE-L genomes compared to between 7.3 and 35.2% in other MRE. In addition to lower pseudogene and repeat content, MRE-L genomes had lower coding density compared to the majority of MRE from other fungal hosts (Fig. 2c). Several MRE genomes, including those of MRE-B, were enriched in content identified as phage. The majority of the top viral hits to genes from identified phage regions were from Caudovirales phage, and phage previously identified in *Spiroplasma*. Many identified phage genes were classified by BLAST as *XerD* site-specific recombinase (Supplementary Data 2).

### Phylogenetic evidence indicates horizontal transfer of host genes to MRE

**Fig. 3:**
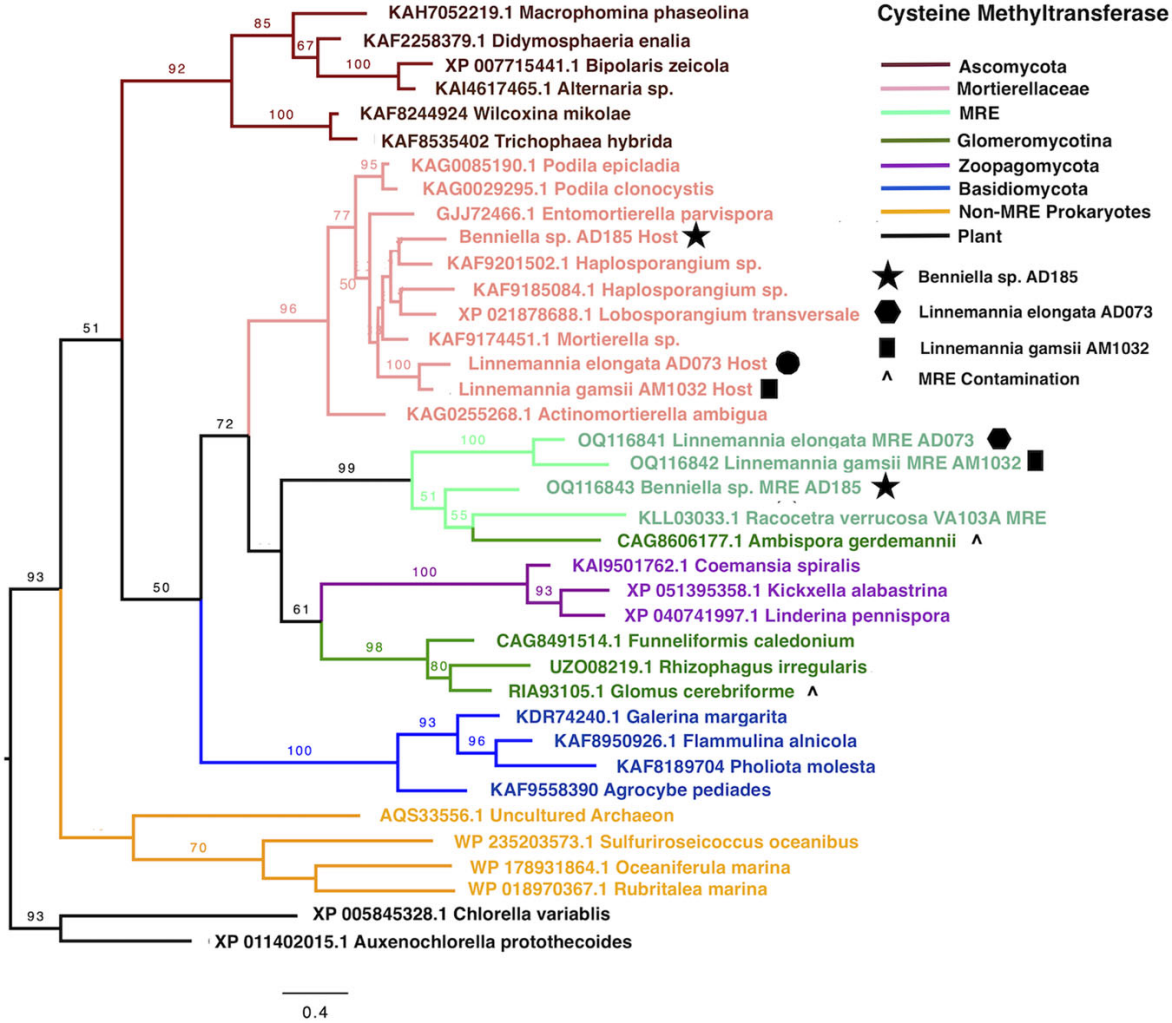
Phylogenetic evidence demonstrates horizontal transfer of the *MGMT* gene between fungal hosts and endobacteria. Maximum likelihood phylogeny of Methylated-DNA-protein-cysteine (*MGMT* gene) from Mortierellaceae MRE and diverse fungal and bacterial BLAST hits. Phylogeny indicates fungal origin of *MGMT* gene in MRE. Bootstrap values (100 replicates) >50 are shown on branches.

Sequence similarity searches indicated that several fungal genes may have been transferred from hosts to MRE. An HGT candidate was identified as a gene encoding a protein cysteine methyltransferase in all Mortierellaceae MRE genomes except *B. erionia* GBAus27B. In each MRE genome, the cysteine methyltransferase genes were flanked by bacterial genes (Supplementary Data 3). Phylogenetic evidence supported HGT of cysteine methyltransferases as those from MRE strains clustered with those from fungal taxa in Glomeromycotina and Zoopagomycota. We note that several Glomeromycotina genome assemblies appeared to be contaminated by MRE sequences (bacterial contigs incorrectly annotated as fungal contigs) (Fig. 3).

Additional HGT gene candidates were also detected throughout various Mortierellaceae MRE assemblies. Several HGT candidate genes encoding *PknD* serine threonine protein kinases were found in both MRE-B. Additionally, *PknD* kinases were found in both MRE-B genomes and *Linnemannia gamsii* AM1032 MRE which only had similarity to sequences from Glomeromycotina and MRE. PknD kinases primarily shared similarity with various kinases, including tyrosine kinases, and proteins containing leucine rich repeats (LRR) in fungi and MRE. MRE PknD proteins formed two clades, with one clade composed of PknD proteins from MRE and Mortierellaceae while a second clade was composed of PknD proteins from MRE and Glomeromycotina fungi. Pknd proteins from *B. erionia* GBAus27B and *Benniella* sp. AD185 were present in both clades. As was found in the cysteine methyltransferase analysis, one of the Glomeromycotina assemblies appeared to be contaminated by MRE (Supplementary Fig. 1). The genes surrounding MRE *PknD* genes were primarily genes of bacterial origin or genes encoding proteins with LRR from Glomeromycotina fungi and other MRE (Supplementary Data 3). Several other genes including those coding for a hypothetical protein, an additional kinase, and a polysaccharide lyase were also potentially horizontally transferred but could not be verified with this type of phylogenetic approach due to lack of homologs or low bootstrap support (Supplementary Data 3).

### Fungal endobacteria display reduced protein lengths

MRE-B, MRE-L, non-*Rhizophagus* AMF, and Endogonales had reduced average protein lengths compared to other Mollicutes (Supplementary Fig. 2). MRE from *Rhizophagus irregularis* genomes did not display similar reductions in protein length and had larger average proteins than *Phytoplasma* and similar average lengths to *Spiroplasma* representatives. BRE also appeared to have reduced average protein sizes compared to non-endofungal *Burkholderia* with an average protein length reduced by 3.1%. The average length of proteins in MRE-L and MRE-B were reduced by 10.8% and 20%, respectively, compared to *Phytoplasma* taxa, which had the smallest average protein lengths among non-MRE Mollicutes. Proteins which were detected as shared single copy orthologs across all MRE or in a single clade of MRE were larger than overall protein distributions (Supplementary Fig. 3). Additionally, annotated proteins were significantly longer in each Mortierellaceae MRE than hypothetical proteins from the same host (Supplementary Fig. 4).

### Degree of genome rearrangement differs by fungal host

**Fig. 4:**
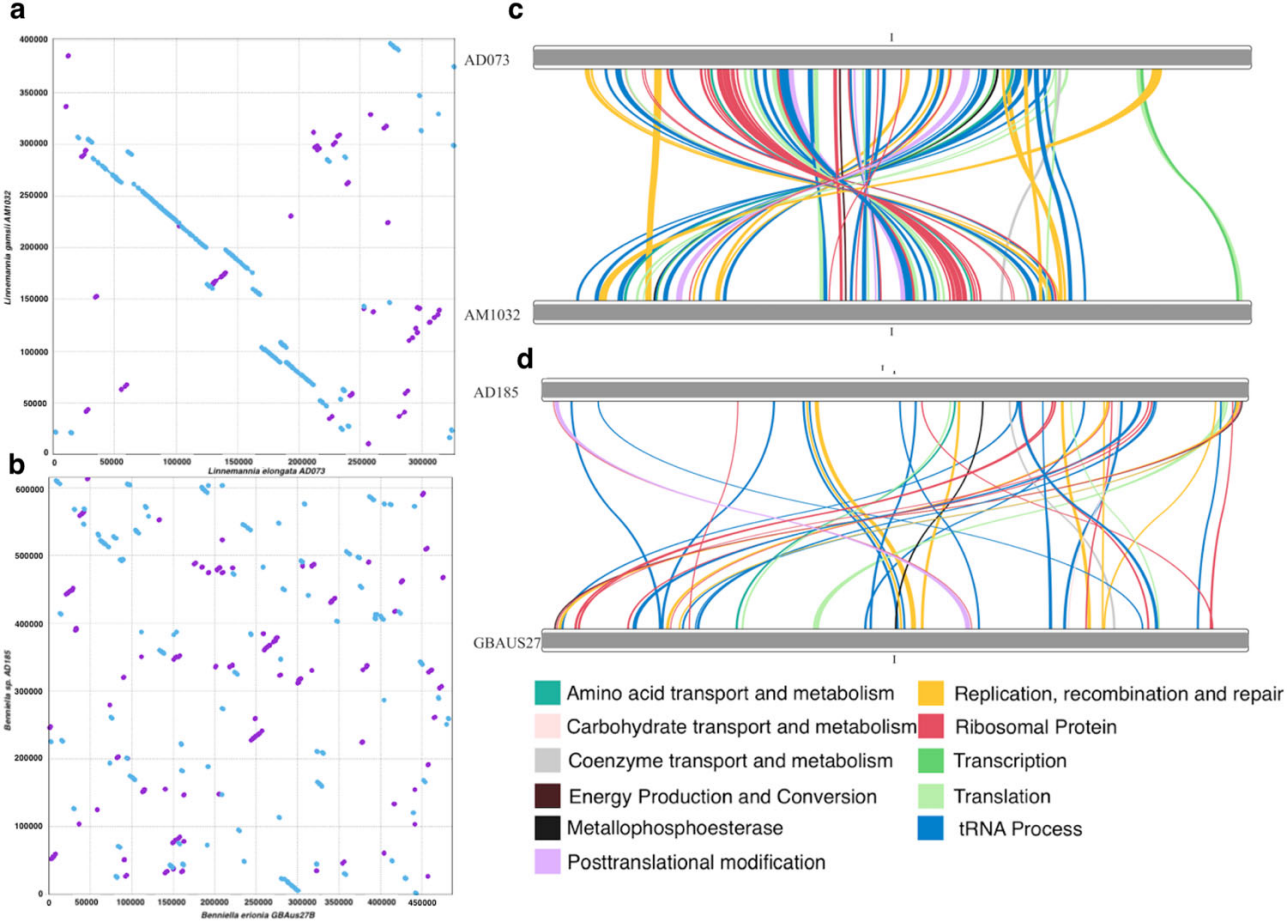
MRE from *Linnemannia* and *Benniella* differ in degrees of genomic rearrangement. Synteny analysis of *Linnemannia* and *Benniella* MRE genomes. Dotplot showing whole genome synteny between (**a**) *Linnemannia* MRE and (**b**) Benniella MRE. Synteny of single copy orthologs identified by BUSCO analysis between (**c**) *Linnemannia* MRE (87 orthologs) and (**d**) *Benniella* MRE (53 orthologs).

Whole genome comparisons with genome alignments (Fig. 4a, b) and localization of conserved BUSCO gene analyses (Fig. 4c, d) demonstrated greater synteny in MRE-L genomes and a greater number of rearrangements in MRE-B genomes. The total length of 1:1 synteny (syntenic regions with a single match in each genome) between MRE-B genomes was 260 kbp compared to 220 kbp between MRE-L genomes. However, the syntenic regions between MRE-B genomes were small and spread sporadically throughout the two genomes whereas the syntenic regions between MRE-L occurred in longer stretches of collinearity (up to 130,000 bp) (Fig. 4a, c). The lack of long syntenic regions and the lack of collinearity between BUSCO orthologs (Fig. 4b, d) indicate a high degree of rearrangement in at least one of the MRE-B genomes.

### Reduced functional capacity of MRE

**Fig. 5:**
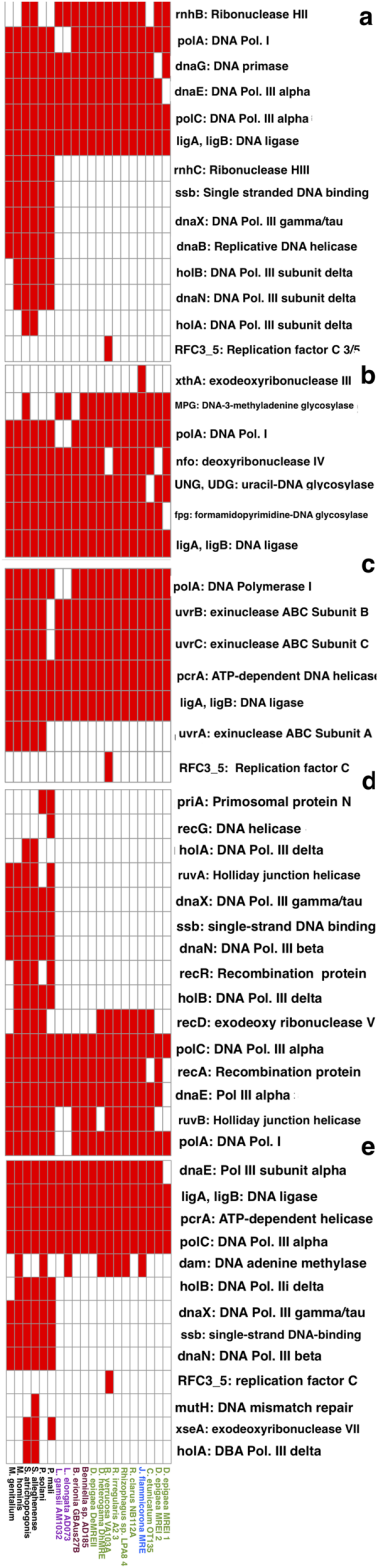
MRE are reduced in gene content related to DNA replication and repair. Functional repertoire of MRE vs selected Mollicutes. Presence (red) / absence (white) heatmaps of genes associated with (**a**) DNA replication, (**b**) Base excision repair, (**c**) Nucleotide excision repair, (**d**) Homologous recombination, (**e**) Mismatch repair.

**Fig. 6:**
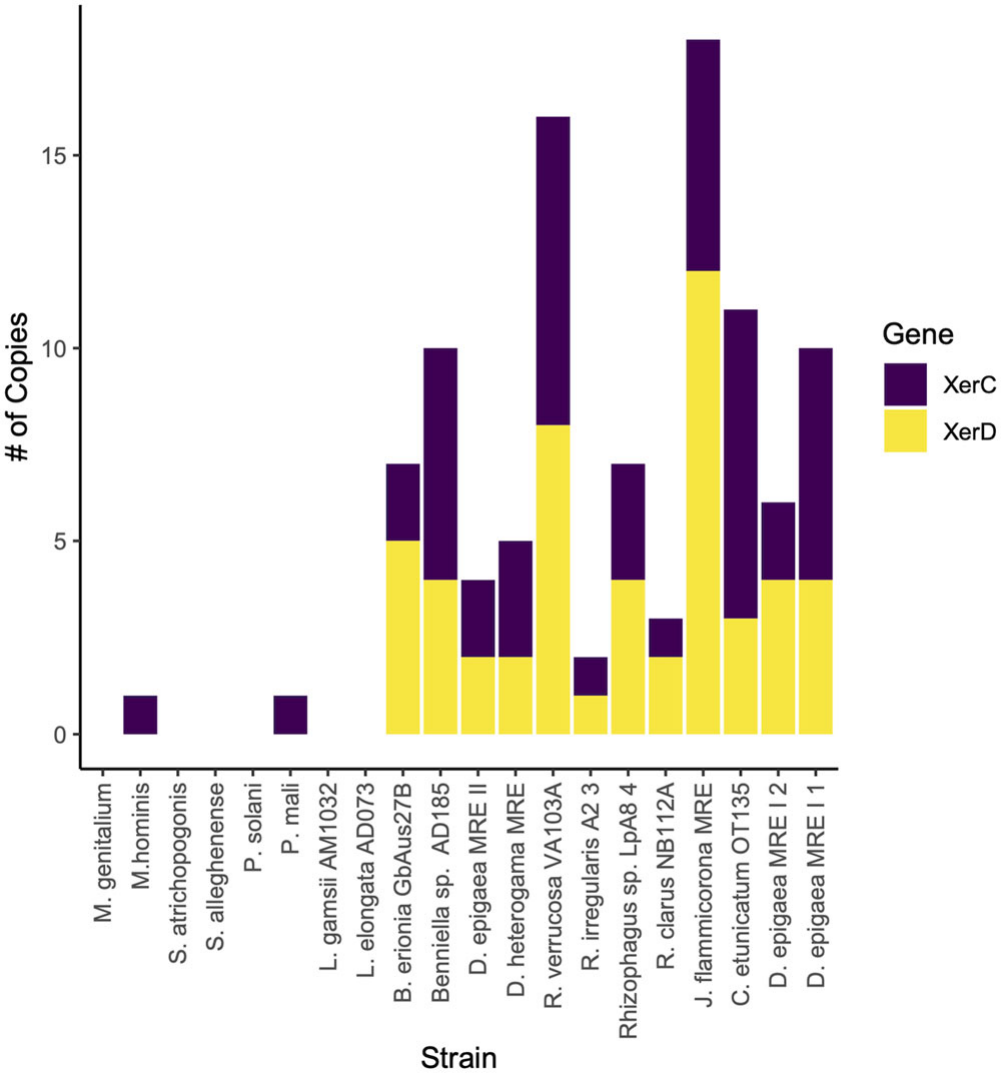
*Linnemannia* MRE lack site-specific recombination genes which are enriched in other MRE genome. Counts of *XerC* and *XerD* genes in MRE and selected Mollicutes genomes.

Metabolism BRITE categories were substantially reduced in all MRE compared to other Mollicutes, particularly in carbohydrate metabolism where MRE genomes contained between zero and four genes related to carbohydrate metabolism compared to between 32 and 144 in other Mollicutes (Supplementary Fig. 5)^27^. Additionally, MRE lacked genes related to membrane transport, with less than three genes per genome compared to 22–63 genes in other Mollicutes. MRE genomes were also reduced in genes related to replication and repair compared to other Mollicutes taxa, particularly in homologous recombination and mismatch repair, and genes encoding three subunits of DNA polymerase III (Fig. 5). Most missing DNA repair genes were lost across all MRE regardless of fungal host, however, at least one copy of genes related to site-specific recombination (*XerC* and *XerD*) were detected in all MRE genomes except for the two MRE-L. *XerC* and *XerD* were also not detected in many of the non-endofungal Mollicutes taxa which were assessed (Fig. 6).

## Discussion

This study presents the first complete, high quality genome assemblies of MRE from cosmopolitan soil fungi in the Mortierellaceae and includes the smallest known fungal endobacterial genome to date (*Linnemannia elongata:* 326,911 bp). We demonstrate for the first time the existence of at least four distinct MRE clades composed of MRE hosted by diverse Mucoromycota fungi. All MRE genomes share several features indicative of long-term evolution within fungal hosts including a near complete loss of carbohydrate metabolism and a reduced capability for DNA repair. These losses make MRE survival outside of fungal hosts or living cells unlikely. Although MRE share similarities, representatives from different fungal hosts vary in genome size, recombination ability, degree of rearrangement, distribution of protein lengths, pseudogene content, and repeat content. These differences may indicate specialization, variable selective pressures between host environments, and/or different timing of host invasion.

As previously reported for MRE taxa from AMF hosts, host phylogeny was a poor predictor of MRE relationships within Mortierellaceae hosts^19^. Phylogenetic comparisons indicated MRE from *Benniella* and *Linnemannia* are more distantly related to each other than are their respective fungal hosts (Fig. 2). Importantly, our results provide insight into the evolutionary history of MRE and provide support for the late invasion hypothesis. The late invasion hypothesis postulates that MRE were acquired through independent lineage-specific invasions, and/or were horizontally transferred between lineages, as opposed to being acquired by a common ancestor and transmitted vertically^23^. Our findings help to resolve the evolutionary history of MRE, as an early invasion would be expected to result in cophylogeny of MRE and their fungal host due to co-evolution alongside fungal hosts, but this was not found.

Although MRE from *Linnemannia* and *Benniella* belonged to separate phylogenetic clades, their genomes contained evidence of shared horizontally transferred genes. Phylogenetic evidence suggests that the *MGMT* gene, encoding a cysteine methyltransferase was transferred from fungi in Glomeromycotina to MRE-L, MRE-B, and AMF MRE. Gene gain in endosymbionts with minimal genome sizes must be adaptive and raises the possibility that this gene is used by MRE to regulate and manipulate their host. Other methyltransferases have been shown to be extensively transferred among archaea and bacteria^28^. As previously reported for AMF-associated MRE, kinase genes and LRR domains have been transferred from fungal hosts to MRE. Kinase genes and LRR domains were transferred into the genomes of many MRE and appeared to be present as multiple copies in some genomes including MRE-B and MRE-L AM1032^13,19,21^. These genes formed two clades, with one clade consisting of genes from MRE that appeared to have been transferred from Mortierellaceae, and another composed of genes transferred from Glomeromycotina (Supplementary Fig. 1). Many of these *PknD* kinases were surrounded by other genes containing LRR domains which had BLAST hits in both MRE and Glomeromycotina, indicating duplications of these genes following HGT. The evolutionary history of proteins containing LRR domains is unclear, but it is thought that HGT may have occurred between eukaryotes and prokaryotes or that there have been multiple origins; additionally, duplications of LRR domains are thought to be common^29,30^.

The presence of shared HGT genes between multiple MRE indicates that these genes were either obtained by an endofungal ancestor of MRE and subsequently lost in some MRE lineages; alternatively, they could have been acquired through multiple independent events. The possibility of HGT in an endofungal ancestor of all MRE does not preclude the late invasion hypothesis as a single MRE ancestor may have acquired the gene and then the MRE may have been horizontally transferred between lineages. Alternatively, the genes may have been acquired through multiple independent events as was demonstrated for horizontal transfer of genes between plant hosts and parasitic plants^31^. *PknD* genes appear to have undergone multiple transfers from fungi to bacteria as genes from the same MRE appear in distinct phylogenetic clades (Supplementary Fig. 1). Multiple independent transfers and maintenance of transferred genes likely implies selection for the maintenance of the genes. For example, as has been proposed in parasitic plants, there may be selection for the transfer and maintenance of genes such as *PknD* kinases containing LRR that may function in controlling host gene expression through microRNAs involved in silencing cascades in plants^32^. It is unclear if the *MGMT* gene may play a role in controlling host gene expression, but maintenance of this gene may be selected for due to its role in DNA repair. Future expression studies will provide insights into the activity and functional roles of horizontally transferred genes.

MRE genomes universally displayed a reduced functional capacity and a near complete loss of genes for energy metabolism and ATP generation, as previously observed in MRE from AMF^13,19,21^. All MRE also had a reduction in genes related to DNA repair, particularly homologous recombination. A loss of genes related to homologous recombination has previously been characterized as a potential feature of vertically transmitted bacterial endosymbionts^3,33–35^. All examined MRE genomes were missing nearly identical sets of DNA repair genes, indicating relaxed selection for this functional group. However, MRE-L genomes were missing several DNA repair genes which were present in most other MRE. These genes included *PolA* (polymerase and exonuclease activity) which was present in all non MRE-L genomes and *RuvB* (holliday junction helicase) which was present in all MRE except for MRE-L and MRE from *D. epigaea*. The loss of these genes is likely to lead to reduced DNA repair capabilities, thus faster evolution, and potentially a greater degree of reliance on fungal hosts^36^.

Although all MRE were missing a similar set of recombination genes, all MRE genomes with the exception of both MRE-L retained *XerC/D* genes associated with site-specific recombination, specifically the resolution of chromosome dimers formed during homologous recombination^37^. In many cases, *XerC/D* were in regions identified as being of potential phage origin, and *XerC/D* genes have been found to be encoded in phage genomes but due to the divergent nature of these genes it is unclear if they are of bacterial or phage origin^38,39^. Phage can also exploit XerC/D recombination systems of their bacterial hosts for integration, creating areas of phage-acquired content around bacterial *XerC/D* genes^38^. However, their presence in identified phage regions and their absence from MRE-L genomes, which also had no observed phage content, suggests a role of the identified recombinases in phage integration. The presence of *XerC/D* in potential phage regions is in agreement with previous hypotheses that phage may play a role in the rearrangement of MRE genomes^13^. Outside of phage integration, in previously sequenced MRE and *Mycoplasma* taxa these genes were thought to be responsible for genome rearrangements and inversions; therefore enhancing genome plasticity^13,37,40^. The lack of these genes and therefore a reduction in rearrangements in MRE-L may also explain the highly distinct differences in observed synteny between MRE-L compared to MRE-B. This was found to be the case for *Carsonella* which had a high degree of synteny between genomes and lacked Xer recombinase systems^41,42^. The increased synteny between MRE-L genomes may indicate that *XerC/D* genes were lost early in MRE-L evolution leading to a lack of rearrangements that characterize other MRE, or the genes were gained from phage in other MRE which never occurred in MRE-L.

MRE-L genomes also displayed other features which make them distinct from MRE in other fungal host lineages. MRE from *Linnemannia* had the smallest genomes and had reduced GC content compared to MRE from other fungal host lineages (Supplementary Data 1). Reduced GC content is a common feature of long-term bacterial endosymbionts of insects and is thought to be the result of a mutational bias toward greater AT content^43,44^. Small genome sizes and reduced GC content suggests that MRE from *Linnemannia* have been endosymbionts for a longer time than other examined MRE. Yet, comparisons of coding density are in disagreement with these observations as MRE-L had relatively low coding density (78.6, 79.2%) compared to most other examined MRE which had coding densities above 80%. Increased coding density is typically thought to be associated with later stage bacterial endosymbionts, but previous deviations have been observed^3,45^. The reduced coding density in MRE-L may also be due to the complete loss of those genes, which have only become pseudogenized in other earlier-stage MRE lineages. These genomic features provide further evidence in support of the late invasion hypothesis as MRE-B taxa have GC contents and coding densities that are more similar to many MRE from AMF than to MRE-L from closely related fungal hosts.

MRE-L also had fewer identified repeat sequences and pseudogenes compared to MRE from other fungal hosts. This reduction, along with general reductions in genome size, is thought to be a feature of long-term endobacterial associates of insects, whereas bacteria that have more recently become host restricted are enriched in mobile genetic elements and pseudogenes^3^. It is possible that the increased pseudogene content reflects the maintenance of recombination genes in non MRE-L genomes, with deleterious genes acquired by horizontal transfer being continuously pseudogenized, as has been previously suggested^37^.

Divergence between MRE from the two Mortierellaceae genera was also reflected in distributions of their predicted protein lengths, with MRE-B proteins being smaller than MRE-L proteins on average. MRE protein lengths were smaller than most other Mollicutes taxa with the exception of *S. atrichopogonis* which had previously been shown to have reduced protein lengths compared to other *Spiroplasma*^46^. These findings support previous observations that orthologous genes tend to be shorter in the insect endosymbiont *Buchnera* compared to free-living bacteria such as *E. coli*^47^. This phenomenon likely occurs due to the accumulation of smaller pseudogenes and the loss of larger genes encoding for larger proteins^48^. Lipman and colleagues also postulated that there is an evolutionary trend towards smaller proteins unless there is a selective functional constraint that requires the length of the protein to be maintained, as is likely to be the case for conserved proteins^49^. Proteins conserved across MRE lineages or MRE phylogenetic clades were on average longer than overall protein repertoires, indicating that these conserved proteins may perform essential roles in bacterial physiology. Additionally, annotated proteins were significantly longer on average than hypothetical proteins, indicating that proteins which maintain well-annotated functions are not shortened whereas nonfunctional proteins or proteins of ambiguous functions likely accumulate deletions and become shorter^49^. This likely indicates that the reduction in protein length is not due to a decrease across all proteins, but instead primarily occurs in proteins without obvious functions. In the case of MRE and other endosymbionts, functional constraints may be relieved by relying on hosts for most functions, thereby allowing for greater reduction in average protein length, numbers of proteins, and total genome size.

In conclusion, examination of MRE genomes from the Mortierellaceae provides evidence of a late invasion of MRE into fungal hosts followed by possible subsequent movement between host lineages. These findings provide valuable insights for continued investigations into how MRE may impact fungal ecology, including larger impacts on microbiome dynamics and plant-fungal interactions in natural environments. Follow-up studies should assess MRE gene expression in order to determine the functional roles of MRE and to assess the degree to which MRE rely on their hosts. The existence of shared horizontally acquired genes by most MRE provides a mechanism for controlling their host and indicates the possibility of a common endofungal ancestor of MRE, which may have subsequently been horizontally transferred between fungal lineages. Future work will assess the expression of genes horizontally transferred from fungal hosts to MRE to uncover the roles of horizontally transferred genes in MRE establishment and biology. Differences in genomic characteristics indicate that MRE have been evolving alongside their hosts for different lengths of time or have been experiencing different selective pressures within their respective hosts. These differences in evolutionary trajectory and degree of genome reduction make MRE an ideal system for studying endosymbiont evolution at different stages of intracellular adaptation. Additionally, the large-scale loss of DNA-repair mechanisms across MRE may be one driver of the notably rapid rate of MRE evolution, making MRE an ideal system for studying evolution in a laboratory setting. The loss of *XerC/D* genes in MRE-L (and the maintenance in MRE-B) will make Mortierellaceae MRE an ideal system for studying the impact of these genes on genome-wide recombination and rearrangement. Future work utilizing long-term and directed evolution should provide valuable insights into the timing of endosymbiotic events across MRE by assessing if *Benniella* MRE genomes become more structurally and functionally similar to *Linnemannia* MRE genomes over time.

## Methods

### Fungal isolate growth, DNA extraction, and MRE confirmation

Four Mortierellaceae isolates known to harbor MRE were utilized in this study. These included *Linnemannia elongata* (AD073), *L. gamsii* (AM1032), *Benniella erionia* (GBAus27B) and an unidentified species of *Benniella* (AD185). Information about these isolates as well as host and MRE genomes are available through JGI Mycocosm^50^. Isolates were grown on 1% malt agar plates for 10 days prior to flash freezing in liquid Nitrogen and grinding with a mortar and pestle. Extraction buffer (containing 0.2 M Tris-HCl, 1 M NaCl, 0.2 M EDTA, and 10% SDS) was added, and samples were incubated at 37C for 30 min with RNAse A (100 ug). Proteinase K (200 ug) was added, and samples were incubated at room temperature for 20 min. A 0.2 volume of 5M Potassium Acetate was added and tubes were chilled on ice for 5 min. After centrifugation (5000 g for 20 min), the aqueous layer was transferred and 0.8 volume of chloroform and isoamyl alcohol (24:1) was added. Samples were centrifuged (15,000 g for 10 min at 4 °C), and the aqueous layer was transferred. This washing step was repeated. A 1 volume cold chloroform was added, and samples incubated for 5 min on ice. Samples were centrifuged (15,000 g, 5 min, 4 °C) and the aqueous layer was transferred. A 0.1 volume 4 M NaCl was added, and samples were inverted 10 times. A 1 volume cold isopropanol was added, and samples were inverted 20 times. Samples were incubated on ice for 10 min then centrifuged (10,000 g for 30 min at 4 °C). Supernatant was decanted and pellet was washed with cold 80% ethanol. Ethanol wash was repeated twice. After final centrifugation, samples were incubated at room temp until all ethanol was evaporated, about 5 min. Samples were eluted in 10 mM TE buffer (pH = 8.0) overnight. Residual contaminants were removed using Cleanup Buffer (2% CTAB, 10 mM Tris, 20 mM EDTA, 1.4M NaCl, 1% PVP, and 0.35 M sorbitol) incubated at room temperature for 10 min then on ice for 5 min. A 1 volume of cold chloroform was added and incubated for 2 min before centrifugation (15,000 g for 10 min at 4 °C). The aqueous layer was removed and washed again with chloroform. A 0.1 volume of 5 M ammonium acetate and 1 volume cold isopropanol was added, and samples were incubated on ice for 10 min before centrifugation (10,000 g for 30 min at 4 °C). Two 80% ethanol washes were performed as before. Pellets were resuspended in TE buffer as before. High molecular weight gDNA purity was assessed using Qubit Fluorometer and shearing was assessed by running samples on 1% agarose gel. Prior to sequencing fungal metagenomes, MRE were confirmed in DNA extracts by amplifying and sequencing 16S rDNA from each isolate with the primers 109F and 1184R^14^ as described by ref. ^12^.

### MRE visualization

Endobacteria were visualized through fluorescence in situ hybridization (FISH) of 16S and 18S rRNA following the fluorescence staining method outlined by ref. ^51^. First, isogenic cultures of *Benniella erionia* GBAus27B including wildtype (carrying MRE) and cured (isolates cured of their MRE with antibiotic treatments) were grown on potato dextrose agar (PDA; BD Difco). A ~7 mm diameter section of mycelium was excised from each isolate after approximately 10 days and transferred to a fixing solution of 4% formaldehyde (ThermoFisher Scientific) in PBS and incubated at 4 °C overnight. Fixed mycelia were then washed 3 times with PBS and treated with a solution containing 1 mg•ml−1 lysozyme (MilliporeSigma), 0.5 mg•ml−1 chitinase (MilliporeSigma), and 5 mg•ml−1 glucanase (MilliporeSigma) for 1 h at 37 °C. Each sample was then dehydrated with a stepwise series of ethanol treatments: 50%, 75%, 100%, 75%, 50%, and finally replaced with PBS for 3 min between each step. Cell wall digested samples were next pre-treated with a 6.5x solution of saline sodium citrate buffer (SSC, ThermoFisher Scientific) supplemented with 0.1 U•ml−1 SUPERase RNase inhibitor (Thermo Fisher Scientific) for 30 min at room temperature. The pre-treatment was then replaced and incubated overnight at 37 °C with a probe hybridization solution containing 125 nM of the 16S universal probe, EUB338 (Cy3-5’-GCTGCCTCCCGTAGGAGT-3’-Cy3), and 18S universal probe, EUK516 (AlexaFluor 647-5’-ACCAGACTTGCCCTCC-3’) in 6.5× SSC (ThermoFisher Scientific) supplemented with 15% formamide (ThermoFisher Scientific)^52^. The samples were then washed with 6.5× SSC + 15% formamide for 5 min at 37 °C a total of 4 times. Samples were washed once more with 5× SSC for 5 min and finally rinsed with 2× SSC twice before transferring to a standard glass slide (VWR) with an inoculating loop and mounted with No. 1.5 coverslip (22 × 22 mm, Corning) using ProLong Glass Antifade mountant (ThermoFisher Scientific). All samples were allowed to cure for approximately 48 h in complete darkness according to the manufacturer’s protocol.

Microscope imaging was performed on an Olympus IX83 with a UPLFL OPH 100 × 1.30 NA oil objective lens. Filter sets for Cy3 and Cy5 were used for excitation and emission of the 16S and 18S probes and visualized through a NA 0.45 20× or NA 1.3 100× objective lens (LUCPLFLN20XPH, UPLFLN100XO2PH). A Marzhauser motorized X/Y stage (48-24-580-0000) was used in combination with the cellSens Dimension Version 3 software to map the fluorescence across the mycelia. The images were saved as a multi-TIFF image containing the X, Y, Z and fluorescent channel dimensions. For ease of visibility the images were cropped and fluorescence channels were overlaid using FIJI (version 2.3.0/1.53f)^53^. To determine that the bacteria were internalized and correlate the intensity of the 16S and 18S relative fluorescence across the Z axis, a custom python script was used to acquire the minimum and maximum pixel intensity for each image set (16S or 18S fluorescence images) and normalize the images to that min and max scale using the Numpy and MatplotLib libraries^54,55^. This script can be found at https://github.com/dmorales003/endobacteria_analysis. Adobe Premier (Version 15.0) was used to generate the panning video to visualize the relative distribution of bacteria across the fungal mycelia.

### Library preparation

All fungal/MRE metagenomes were prepared for sequencing using Pacific Biosciences (PacBio) library preparation. The details of library preparation for each individual isolate are described below.

#### *Linnemannia elongata* AD073 and *Benniella* sp. AD185

First, 4–5 ug of unsheared genomic DNA was treated with exonuclease and DNA damage repair mix to remove single stranded ends. Ends were then repaired, and blunt universal adapters were ligated using SMRTbell Template Prep Kit 1.0 (Pacific Biosciences). Following ligation of adapters, libraries were purified with AMPure PB beads (Pacific Biosciences).

#### *Linnemannia gamsii* AM1032

Due to the suspected presence of contaminants, 6.8 ug of genomic DNA was pre-cleaned with a salt-chloroform wash. Cleaned DNA was then treated with exonuclease and DNA damage repair mix to remove single stranded ends. Ends were then repaired, and blunt universal adapters were ligated using SMRTbell Template Prep Kit 1.0 (Pacific Biosciences) The library was then purified with AMPure PB beads (Pacific Biosciences). Due to remaining fragments below 2 kb, the cleaned library was size selected for fragments of 6 kb or longer with the BluePippin system (Sage Science).

#### *Benniella erionia* GBAus27B

5 ug of genomic DNA was sheared to >10 kb using Covaris g-tubes. The sheared DNA was then treated with exonuclease and DNA damage repair mix to remove single-stranded ends followed by end repair and blunt end repair and ligation of blunt adapters using SMRTbell Template Prep Kit 1.0 (Pacific Biosciences). Next, the library was purified with ampure PB beads.

### Sequencing

All fungal/MRE metagenomes were sequenced using Pacific Biosciences (PacBio). The details of sequencing for each individual isolate are described below.

#### *Linnemannia elongata* AD073, *L. gamsii* AM1032, *Benniella* sp. AD185

To prepare libraries for sequencing, a PacBio Sequencing primer was annealed to the SMRTbell template libraries and sequencing polymerase was bound to them using Sequel Binding kit 2.1. SMRTbell template libraries were then sequenced on a Pacific Biosystems Sequel sequencer using v3 sequencing primer, 1 M v2 SMRT cells, and Version 2.1 sequencing chemistry with 1 × 600 sequencing movie run times. For AD073, and additional SMRT cell was sequenced with a 1 × 360 movie run time to assess optimal sequencing concentration.

#### *Benniella erionia* GBAus27B

To prepare for sequencing, the PacBio sequencing primer was annealed to the SMRTBell library and a P6 sequencing polymerase was bound. The libraries were then sequenced on a Pacific Biosciences RSII sequencer using Version C4 chemistry and 1 × 240 sequencing movie run times.

### Genome assembly and MRE separation

Following sequencing, sequence data was processed by the JGI QC pipeline (genome.jgi.doe.gov/lookup?keyName=jgiProjectId&keyValue=1103405) to remove artifacts. Filtered read data was then assembled with Falcon to generate initial assemblies^56^. MRE contigs were then identified from each initial host assembly using a combination of BLAST to the NCBI nt database, mapped read coverage, tetranucleotide frequency PCA, and GC content analysis. Assembled MRE content was improved by recruiting error-corrected Falcon pre-assembled reads (preads) or CCS reads using BBTools bbduk.sh [*k* = 31 mm = f mkf = 0.05]^57^. Recruited reads were then assembled. Multiple rounds of read recruitment and reassembly and/or improvement with additional tools were necessary to generate the final assembly in some cases. Final assemblies were then linearized and polished using the raw PacBio data. Following separation of MRE contigs, the fungal genomes of *L. elongata* AD073 and *L. gamsii* AM1032 were assembled using Flye version 2.7.1-b1590 and Flye version 2.5, respectively^58^. Each assembly was polished using either gcpp version SMRTLINK v8.0.0.80529 (*L. elongata* AD073) or Arrow version 7.0.1.66975 (*L. gamsii* AM1032). *Benniella* sp. AD185 was assembled using Falcon version pb-assembly 0.0.2|falcon-kit = 1.2.3|pypeflow = 2.1.0, improved using finisherSC v2.1 and polished using Arrow version SMRTLink v6.0.0.47841^59^. Summary statistics of fungal genome assemblies are available in Supplementary Table 1. Assembly details unique to each individual MRE are available below.

#### *Linnemannia elongata* AD073

An initial Falcon assembly for host and bacterium was performed with the following conditions: version [pb-assembly 0.0.2|falcon-kit = 1.2.3|pypeflow = 2.1.0]^56^ Next, CCS reads were generated using pbccs version 4.2.0 (commit v4.2.0) [--min-rq .99 --min-passes 3] and assembled with Flye version 2.8.3-b1695 [--pacbio-hifi --meta]^58^. A single contig was identified as MRE, this assembly was improved by recruiting CCS reads to the putative MRE contig with BBtools version 38.79[bbduk.sh outm *k* = 31 mm = f mkf = 0.05]^57^. Next, the MRE was reassembled with Flye version 2.8.3-b1695 [--pacbio-hifi]^58^, and finally polished with gcpp version SMRTLINK v8.0.0.80529 [--algorithm arrow] (https://www.pacb.com/support/software-downloads).

#### *Linnemannia gamsii* AM1032

Filtered subread data was assembled together with Falcon version pb-assembly 0.0.2|falcon-kit = 1.2.3|pypeflow = 2.1.0 to generate an initial assembly^56^.The Falcon pre-assembled reads (preads) were recruited to putative MRE contigs using BBtools version 38.75 [bbduk.sh *k* = 31 mm = fmkf = 0.05] and assembled with Flye version 2.5 [--pacbio-corr -g 750 k --asm-coverage 50]^57,58^. This assembly was then improved with finisherSC version 2.1^59^. Several iterative rounds of read recruitment, assembly, and improvement were performed until a circular, 402 kb contig identified as MRE was obtained. The final MRE assembly was then polished with Arrow version SMRTLink v7.0.1.66975 (https://www.pacb.com/support/software-downloads).

#### *Benniella* sp. AD185

Following sequencing, an initial assembly was obtained with Falcon [pb-assembly 0.0.2|falcon-kit = 1.2.3|pypeflow = 2.1.0]^56^. The fungal mitochondria was assembled separately from the Falcon pre-assembled reads (preads) using an in-house JGI tool, assemblemito.sh. Following mitochondrial assembly, it was determined using BLAST that the putative mitochondrial assembly represented the MRE genome. The MRE assembly was then improved to make a single contig with two rounds of read recruitment (BBTools bbduk.sh [mm=f mkf=0.05]) and reassembly with Flye version 2.4 ([--pacbio-corr --genome-size100 K -t 32 --asm-coverage 50])^57,58^. This assembly was linearized and polished with Arrow (https://www.pacb.com/support/software-downloads).

#### *Benniella erionia* GBAuS27B

An initial assembly of host and MRE was obtained with Falcon V 0.4.2^56^. The MRE was separated from the host based on coverage, GC content, and tetramer content. Two rounds of read recruitment and reassembly were performed with Bbtools version 38.79 (*k* = 31 mm = f mkf = 0.05) and Celera version 8.3 under default parameters^57,60^. The resulting assembly was improved with finisherSC version 2.0, and polished with Quiver version smrtanalysis_2.3.0.140936.p5 (https://www.pacb.com/support/software-downloads/)^59^.

### Genome annotation

Prior to annotation, all MRE genomes and others used for comparison (Supplementary Data 1) were assessed for completeness and contamination using the CheckM tool via the KBase web interface^24,61^. Each MRE assembly was annotated with the Prokka annotation tool via the KBase web interface using the NCBI translation table 4 (Mold, Protozoan, and Coelenterate Mitochondrial Code and the *Mycoplasma*/*Spiroplasma* Code) with an E-value threshold of 0.01^61–63^. Additional non-MRE bacterial genomes used in phylogenetic analyses and other comparisons were obtained from the NCBI RefSeq database, the PATRIC database, and the European Nucleotide Archive^64–66^. In addition to annotating novel MRE genomes, all bacterial genomes utilized for comparisons in this study were re-annotated with Prokka to avoid biasing results based on differences in annotation tools. CDS counts, GC content and protein lengths were all obtained from the Prokka results for each assembly. RASTtk was utilized with the *Mycoplasma* genetic code on the KBase web interface to identify repeat regions^61,67^. The Pseudofinder tool was utilized to identify pseudogenes in Prokka annotations using a DIAMOND formatted Swiss-Prot curated protein database^68–70^. PhiSpy was used to identify phage sequences with default settings^71^. Proteins in PhiSpy identified phage regions were considered to be of potential phage origin if they had hits against the NCBI viral database with e values below 1e-5 and query coverages of 50% or more. Prokka predicted proteins were then assigned KEGG categories and placed into BRITE hierarchies using GhostKoala^27,72^. Density plots showing protein length distributions, coding density, and summaries of Pseudofinder, RASTtk, and PhiSpy outputs were created in the *ggplot2* package of R^73^. Fungal genomes were annotated using the JGI annotation pipeline^50^. All fungal genome data are available through the JGI MycoCosm web portal (https://mycocosm.jgi.doe.gov/).

### Horizontal gene transfer detection

Evidence of horizontal transfer between fungal hosts and MRE was identified following methods adapted from ref. ^19^. Briefly, annotated MRE proteins were BLASTed against the NCBI non-redundant protein database using blastp against a maximum of 500 sequences. Hits with E-values below 1e-5 and query coverages above 50% were considered valid. Genes were considered to be HGT candidates if the query returned more than 2X fungal hits than bacterial hits. Queries with fungal hits only in Glomeromycotina taxa were not considered as HGT events as these may represent MRE or other bacteria not identified in initial sequencing of the fungi. However, *pknD* genes containing LRR which only had fungal hits in Glomeromycotina were included in phylogenetics as these genes have previously been identified as being HGT candidates in MRE^19^. Genes passing BLAST filters were then placed into phylogenies including diverse fungal and non-metagenome bacterial/archaeal sequences identified from 1000 blast hits produced from the search of each HGT candidate. Phylogenies were inferred through RAxML with the option to allow automatic selection of the best protein substitution model (-m PROTGAMMAAUTO) and 100 bootstrap replicates^74^. Following the construction of phylogenies, fungal taxa which were found to group with MRE were examined for potential MRE contamination in fungal assemblies by visualizing contigs with VizBin^75^. Contigs which clustered separately from the majority of fungal contigs were blasted against the NCBI non-redundant protein database, and taxa which produced BLAST hits to MRE were identified on phylogenies as having contamination.

### Ortholog analysis and phylogenetics

Ortholog analysis was performed using OrthoFinder on predicted protein sequences^76^ from the four Mortierellaceae MRE, 9 previously sequenced MRE taxa, and two representatives of both *Spiroplasma* and *Phytoplasma* (17 total taxa)^11,13,19,21,22^. Accessions and references for all taxa utilized in these analyses are available in Supplementary Data 1. Only one representative of the three Endogonales MRE genomes was included in phylogenetic reconstructions due the presence of high levels of contamination detected in the other two genomes; the three *Rhizophagus* sp. replicates were also excluded from phylogenetic analysis due to high levels of contamination as detected by CheckM (i.e., duplications of single copy marker genes in MRE genomes, reflecting the presence of multiple genomes) (Supplementary Data 1). Only a single representative of the three *Rhizophagus irregularis* MRE was included as the three genomes were sequencing replicates^22^. Phylogenies were created using RAxML with the option to allow automatic selection of the best protein substitution model (-m PROTGAMMAAUTO) and 100 bootstrap replicates^74^. Phylogenetic trees were visualized with FigTree V1.4.4 (http://tree.bio.ed.ac.uk/software/figtree/). The fungal host phylogeny was created from sequences of beta-tubulin proteins. Information on host beta-tubulin sequences used in this analysis is available in Supplementary Table 3.

### Synteny analysis

Synteny between the two *Linnemannia* MRE and two *Benniella* MRE was analyzed through comparisons of six frame amino acid translation of genomes with the *Promer* function of MUMmer 3 and synteny results were visualized as dot plots using mummerplot^77^. To visualize synteny between shared orthologs, a Benchmarking Universal Single-Copy Orthologs (BUSCO) analysis was performed with a Mollicutes database and BUSCOs shared between both *Linnemannia* or both *Benniella* were visualized to indicate their positions in each genome with the R package *RIdeogram*^26,78^.

### Statistics and reproducibility

Reproducibility of phylogenetic and comparative genomics analyses was ensured by utilizing all publicly available MRE genomes and high-quality genomes of other Mollicutes references. Additionally, reproducibility of phylogenies was ensured by using 100 bootstrap replicates in each phylogeny. When comparing hypothetical and annotated protein lengths, means were compared using a wilcoxon test with a bonferroni multiple comparison correction. Numerical data underlying figures is available in Supplementary Data 4.

### Reporting summary

Further information on research design is available in the Nature Portfolio Reporting Summary linked to this article.

### Supplementary information

Supplementary Information

Description of Additional Supplementary Files

Supplementary Data 1

Supplementary Data 2

Supplementary Data 3

Supplementary Data 4

Supplemental Movie 1

Reporting Summary

## Data Availability

Novel MRE genome assemblies and annotations are available on NCBI under accessions: CP125274 CP125277. All other data are available from JGI MycoCosm: https://mycocosm.jgi.doe.gov/mycocosm/home.

## Code availability

Code for analyzing FISH imaging data is available at https://github.com/dmorales003/endobacteria_analysis.
