# Supplementary material for: Comparative genomics of Mollicutes-related endobacteria supports a late invasion into Mucoromycota fungi
Source: Commun Biol. 2023 Sep 18;6:948. doi: 10.1038/s42003-023-05299-8 (PMC10507103; doi:10.1038/s42003-023-05299-8)
Supplement: Supplementary file 1 — Supplementary Information [file 42003_2023_5299_MOESM1_ESM.pdf]

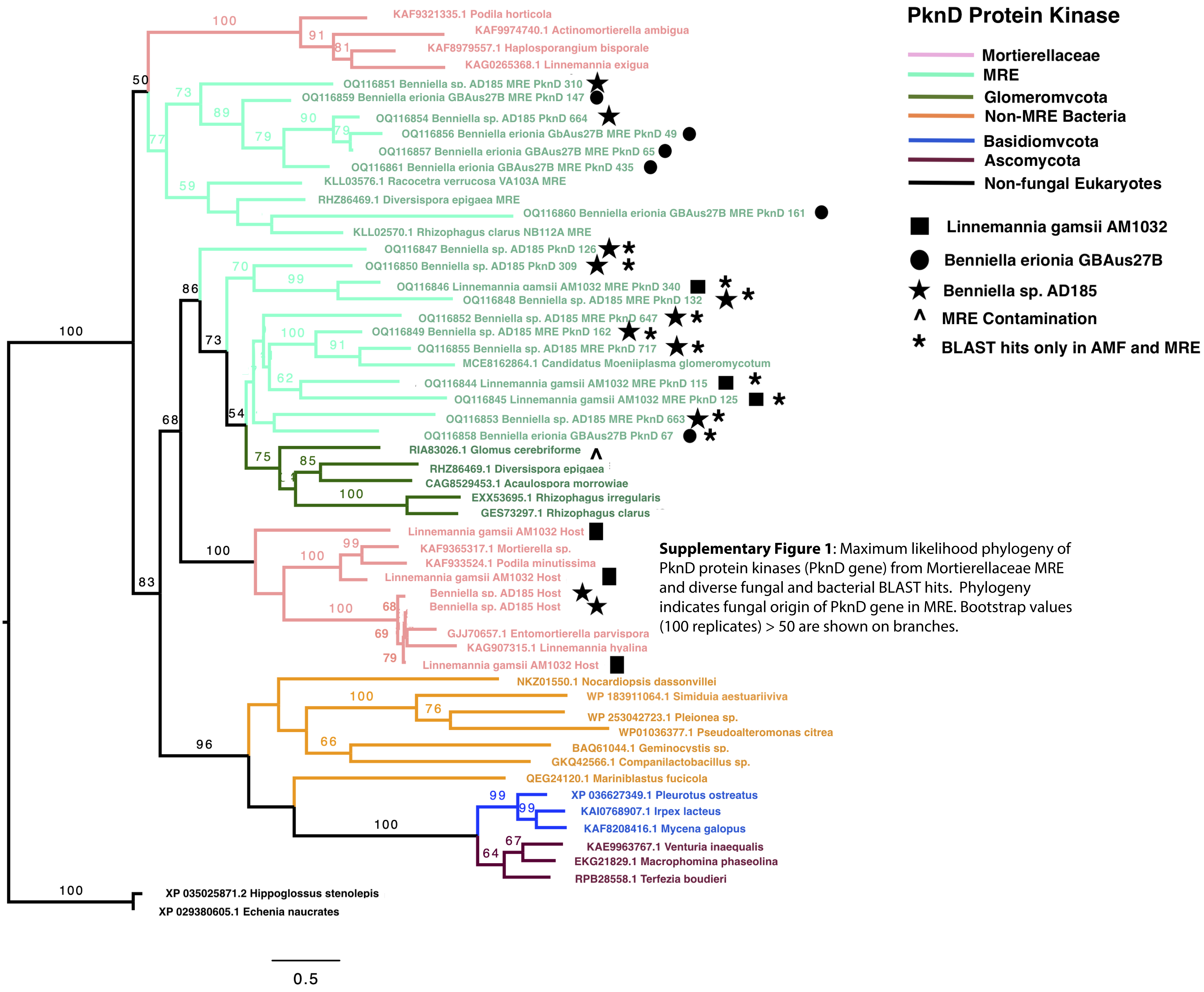

Supplementary Figure 1: Maximum likelihood phylogeny of PknD protein kinases (PknD gene) from Mortierellaceae MRE and diverse fungal and bacterial BLAST hits. Phylogeny indicates fungal origin of PknD gene in MRE. Bootstrap values (100 replicates) > 50 are shown on branches.

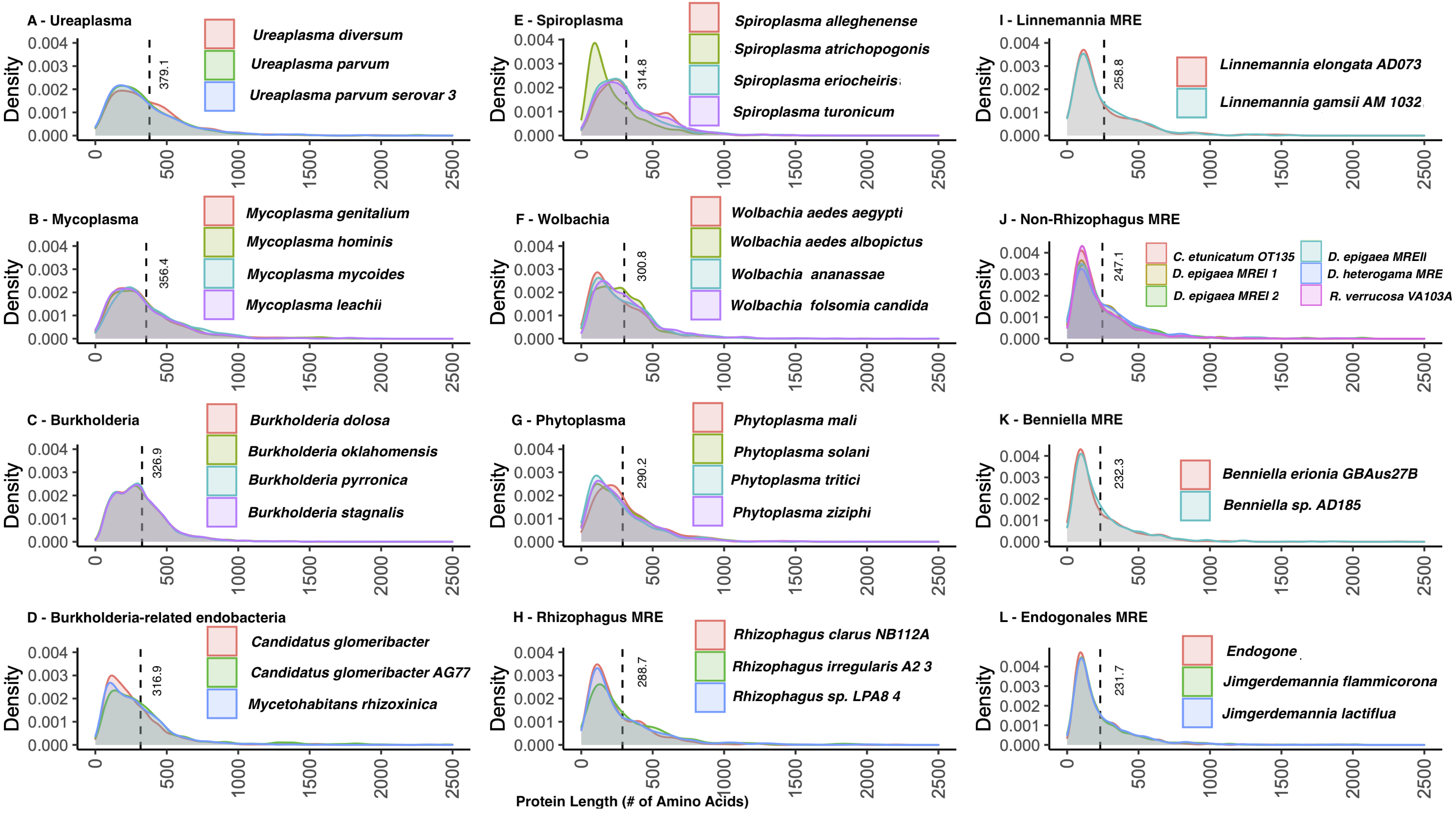

Supplementary Figure 2: Density plots showing protein length distributions protein lengths of selected (A) Ureaplasma, (B) Mycoplasma, (C) Burkholderia, (D) Spiroplasma (E) Burkholderia-related endobacteria, (F) Wolbachia, (G) Phytoplasma, (H) Rhizophagus MRE, (I) Linnemannia MRE, and (J) Non Rhizophagus AMF MRE, (K) Benniella MRE, (L) Endogonales MRE. Distributions indicate that MRE, regardless of fungal host, have reduced average protein lengths and protein length distributions skewed towards smaller proteins compared to other endosymbionts and free-living bacteria. The dashed line represents the average protein length within each genus.

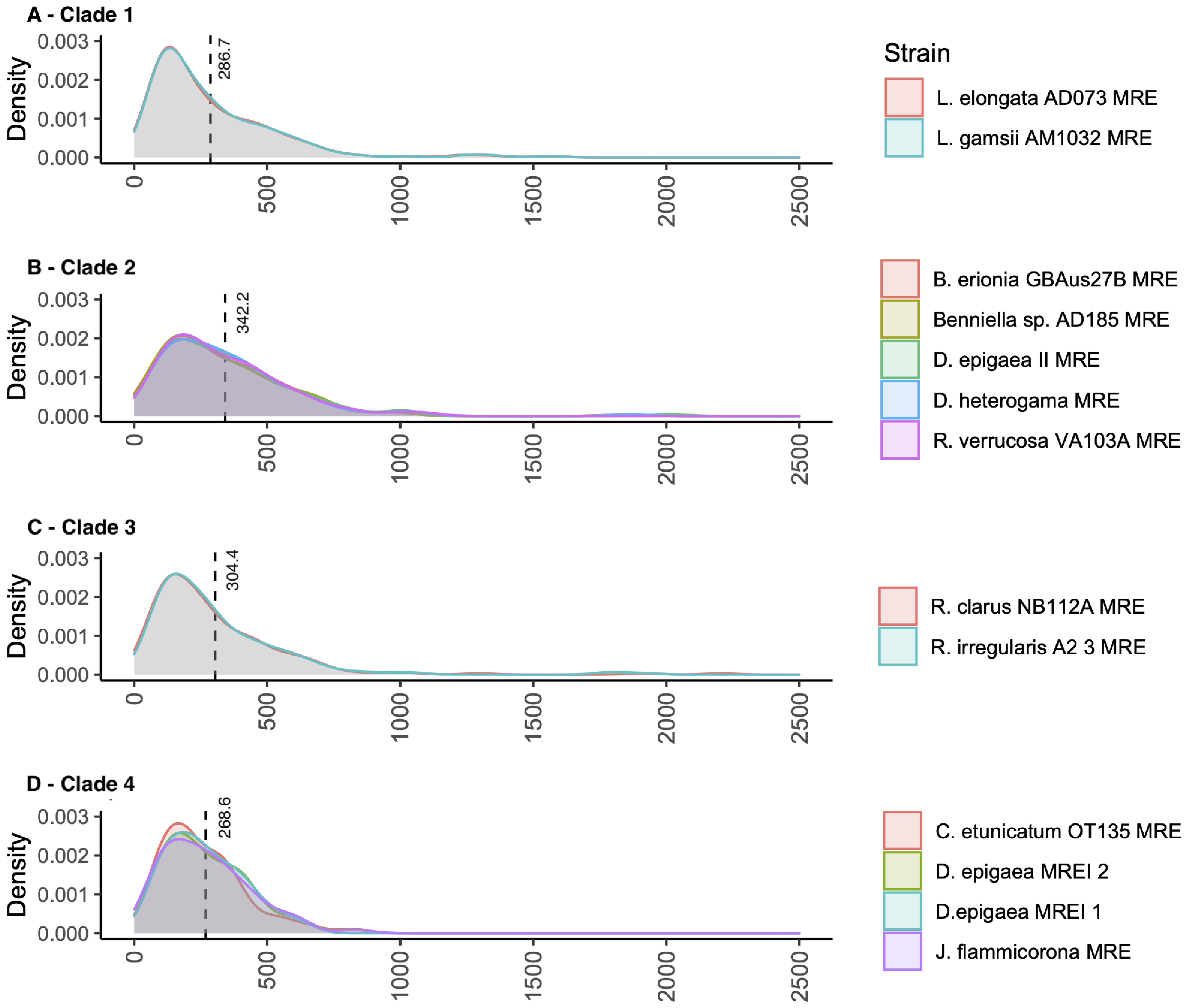

Supplementary Figure 3: Density plots showing protein length distributions of proteins present as single copy orthologs in (A) MRE in clade 1, (B) MRE in clade 2, (C) MRE in clade 3, and (D) MRE in clade 4. Dashed line represents the average protein length for the clade.

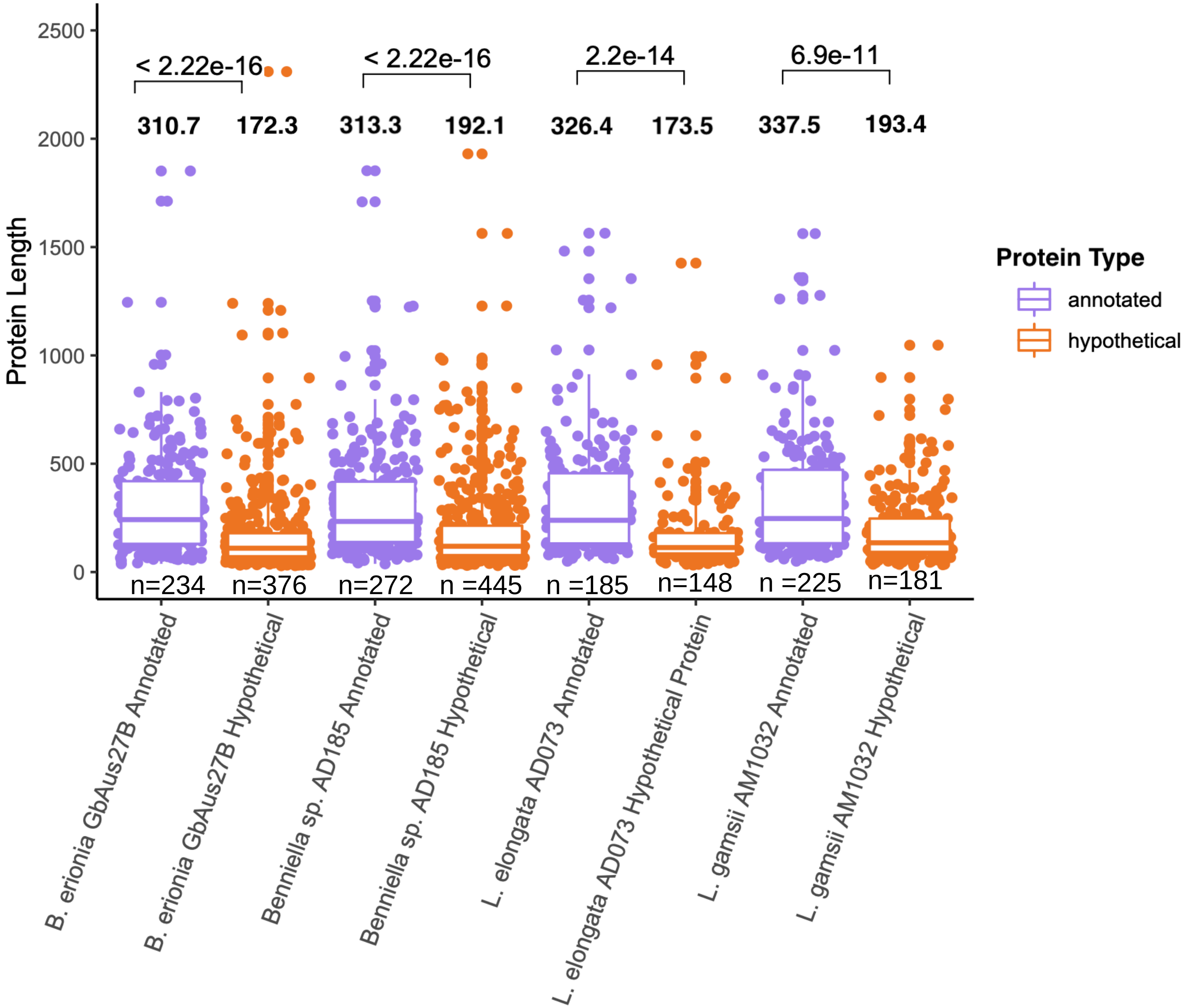

Supplementary Figure 4: Boxplots showing lengths of annotated vs. hypothetical proteins for each of the four Mortierellaceae MRE. Top and bottom lines of the box represent the interquartile range (IQR) and the whiskers represent +/- 1.5* the IQR. Means were compared using a wilcoxon test with a bonferroni multiple comparison correction.

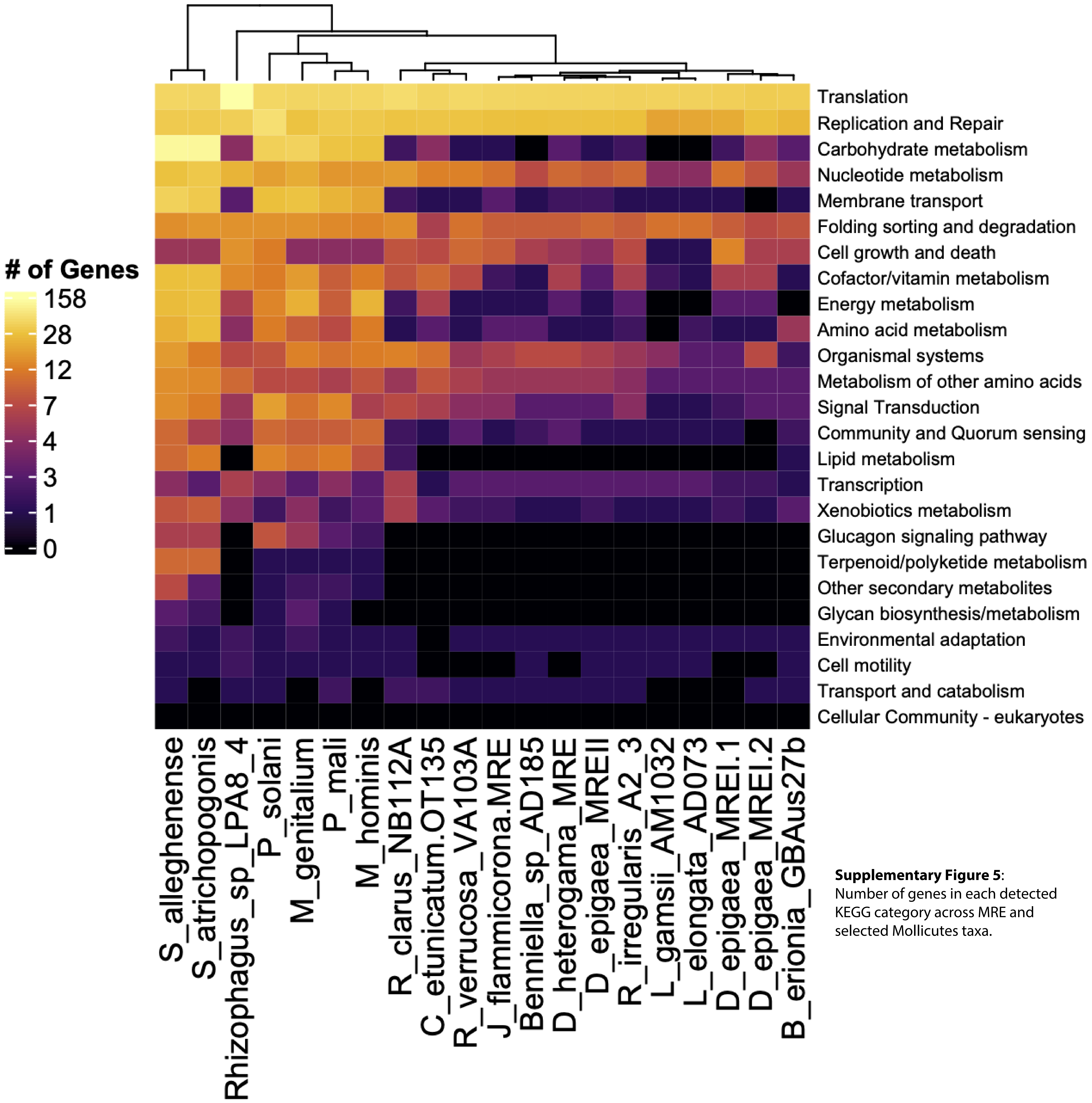

Supplementary Figure 5: Number of genes in each detected KEGG category across MRE and selected Mollicutes taxa.

	L. gamsii AM1032	L. elongata AD073	Benniella sp. AD185	B. erionia GBAus27B
JGI Project ID	1153709	1264458	1103394	1203124
Assembly size (Mb)	57.79	48.2	55.63	44.97
# Scaffolds	324	156	51	140
N50 (Mb)	0.39	0.71	2.64	0.82
% Repeat masked	14.45%	6.28%	5.57%	10.66%
# Genes	14981	13851	15422	13953
# Unique PFAMs	3402	3456	3470	3431
% Genes w/ PFAMs	58.55%	61.97%	59.93%	57.11%
% CDS complete	93.67%	94.49%	93.25%	95.08%
BUSCO* present	98.80%	98.10%	98.50%	97.50%
BUSCO single copy	95.90%	94.70%	95.50%	94.40%
BUSCO duplicated	2.30%	2.00%	2.30%	2.40%
BUSCO fragmented	0.60%	1.40%	0.70%	0.70%

Supplementary Table 1. Assembly and annotation statistics for fungal genomes sequenced as a part of this study, as well as previously published B. erionia GBAus27B. *BUSCO v5.0.0 (Manni et al., 2021) was used to explore annotation completeness by searching against the Mucoromycota ortholog database.

Ortholog Number	Annotated Function
1	Cysteine tRNA Ligase
2	30S Ribosomal Protein S15
3	50S Ribosomal Protein L17
4	ATPase
5	Leucine tRNA Ligase
6	GTPase Der
7	DNA ligase
8	Ribosomal RNA small subunit methyltransferase H
9	Histidine tRNA Ligase
10	Ribosomal RNA small subunit methyltransferase A

Supplementary Table 2: Annotations of single copy orthologs used to create bacterial phylogeny in Figure 2A.

Fungus Name	NCBI Accession Number
Claroideoglomus etunicatum	ACL36840.1
Jimgerdemannia flammicorona	RUS33079.1
Diversispora epigaea	ACL36846.1
Rhizophagus clarus	ACL27209.1
Dentiscutata heterogama	ACL36877.1
Racocetra verrucosa	ACL36830.1
Linnemannia elongata	NA
Linnemannia gamsii	NA
Benniella erionia	NA
Benniella sp.	NA
Rhizophagus irregularis	XP 025185290.1
Neurospora crassa	AAA33617.1

Supplementary Table 3: Sources of beta-tubulin sequences for fungal host phylogeny (Figure 2D)
