# Supplementary material for: Comparative genomics of Mollicutes-related endobacteria supports a late invasion into Mucoromycota fungi
Source: Commun Biol. 2023 Sep 18;6:948. doi: 10.1038/s42003-023-05299-8 (PMC10507103; doi:10.1038/s42003-023-05299-8)
Supplement: Supplementary file 2 — Description of Additional Supplementary Files [file 42003_2023_5299_MOESM2_ESM.pdf]

Description of Additional Supplementary Files

File name: Supplemental Movie 1
Description: A panning fluorescence map of the 16S and 18S rRNA found in GBAus27B visualized by FISH imaging shows the ubiquitous distribution of bacteria across the fungal mycelia.

File name: Supplementary Data 1
Description: Summary statistics of bacterial genomes compared in this study.

File name: Supplementary Data 2
Description: BLAST identification of genes in putative phage regions.

File name: Supplementary Data 3
Description: Horizontal gene transfer candidates. Counts of BLAST hits in bacteria, archaea, fungi, eukaryotes, and viruses for HGT genes, and genes up and downstream of HGT genes.

File name: Supplementary Data 4
Description: Numerical data for Figures 2, 5, 6 and supplementary figures 2, 3, 4, and 5.
