# Supplementary material for: Comparative genomics of Mollicutes-related endobacteria supports a late invasion into Mucoromycota fungi
Source: Commun Biol. 2023 Sep 18;6:948. doi: 10.1038/s42003-023-05299-8 (PMC10507103; doi:10.1038/s42003-023-05299-8)
Supplement: Supplementary file 8 — Reporting Summary [file 42003_2023_5299_MOESM8_ESM.pdf]

nature portfolio

Corresponding author(s): Gregory Bonito
Last updated by author(s): Jul 11, 2023

Reporting Summary

Nature Portfolio wishes to improve the reproducibility of the work that we publish. This form provides structure for consistency and transparency in reporting. For further information on Nature Portfolio policies, see our Editorial Policies and the Editorial Policy Checklist.

Statistics

For all statistical analyses, confirm that the following items are present in the figure legend, table legend, main text, or Methods section.

n/a	Confirmed	
☐	☒	The exact sample size (*n*) for each experimental group/condition, given as a discrete number and unit of measurement
☒	☐	A statement on whether measurements were taken from distinct samples or whether the same sample was measured repeatedly
☐	☒	The statistical test(s) used AND whether they are one- or two-sided<br>*Only common tests should be described solely by name; describe more complex techniques in the Methods section.*
☒	☐	A description of all covariates tested
☐	☒	A description of any assumptions or corrections, such as tests of normality and adjustment for multiple comparisons
☒	☐	A full description of the statistical parameters including central tendency (e.g. means) or other basic estimates (e.g. regression coefficient) AND variation (e.g. standard deviation) or associated estimates of uncertainty (e.g. confidence intervals)
☒	☐	For null hypothesis testing, the test statistic (e.g. *F*, *t*, *r*) with confidence intervals, effect sizes, degrees of freedom and *P* value noted<br>*Give P values as exact values whenever suitable.*
☒	☐	For Bayesian analysis, information on the choice of priors and Markov chain Monte Carlo settings
☒	☐	For hierarchical and complex designs, identification of the appropriate level for tests and full reporting of outcomes
☒	☐	Estimates of effect sizes (e.g. Cohen's *d*, Pearson's *r*), indicating how they were calculated

Our web collection on statistics for biologists contains articles on many of the points above.

Software and code

Policy information about availability of computer code

Data collection	No software or custom code was used in the data collection performed in this study.
Data analysis	Assemblies were performed using Falcon version pb-assembly 0.0.2 and Flye version 2.7.1-b1590 . These assemblies were then polished and improved using BBTools, gcpp version SMRTLINK v8.0.0.80529, and Arrow version 7.0.1.66975.Data analysis was performed using publicly available packages available via kbase (https://www.kbase.us/) Including Prokka (v1.14.5), RASTtk (v1.073), and CheckM (v1.0.18). Additionally, publicly available R packages such as ggplot2 (V 3.4.2) and RIdeogram (V0.2.2) were utilized for vizualizations and statistical analysis. Other analyses were performed using publicly available conda packages such as Raxml (v8.2.12), Orthofinder (v2.2.7), Phispy (v4.2.21), and Busco (v5.4.7).. Other analyses were performed using publicly available tools including figtree (v1.4.4), pseudofinder (v1.1.0 https://github.com/filip-husnik/pseudofinder), and Vizbin (v1.0.0). Kegg analyses were performed using the GhostKoala tool available online (https://www.kegg.jp/ghostkoala/). FISH data analysis was performed using custom code available at: https://github.com/dmorales003/endobacteria_analysis.

For manuscripts utilizing custom algorithms or software that are central to the research but not yet described in published literature, software must be made available to editors and reviewers. We strongly encourage code deposition in a community repository (e.g. GitHub). See the Nature Portfolio guidelines for submitting code & software for further information.

Data

Policy information about availability of data

All manuscripts must include a data availability statement. This statement should provide the following information, where applicable:

- Accession codes, unique identifiers, or web links for publicly available datasets
- A description of any restrictions on data availability
- For clinical datasets or third party data, please ensure that the statement adheres to our policy

MRE genome assemblies and annotations are available on NCBI under accession numbers CP125274-CP12577.

Research involving human participants, their data, or biological material

Policy information about studies with human participants or human data. See also policy information about sex, gender (identity/presentation), and sexual orientation and race, ethnicity and racism.

Reporting on sex and gender	NA
Reporting on race, ethnicity, or other socially relevant groupings	NA
Population characteristics	NA
Recruitment	NA
Ethics oversight	NA

Note that full information on the approval of the study protocol must also be provided in the manuscript.

Field-specific reporting

Please select the one below that is the best fit for your research. If you are not sure, read the appropriate sections before making your selection.

☐ Life sciences ☐ Behavioural & social sciences ☒ Ecological, evolutionary & environmental sciences

For a reference copy of the document with all sections, see nature.com/documents/nr-reporting-summary-flat.pdf

Ecological, evolutionary & environmental sciences study design

All studies must disclose on these points even when the disclosure is negative.

Study description	This study was performed as a comparative genomics study on Mollicutes-related endobacteria with four novel MRE from Mortierellaceae compared to each other and to publicly available MRE genomes. The four novel MRE genomes were selected to represent two genera: Benniella and Linnemannia to allow for cross-genus comparison.
Research sample	The research samples in this study were Mortierellaceae fungal cultures containing endobacteria
Sampling strategy	Samples were selected to represent two genera of fungal hosts following culturing efforts to culture Mortierellaceae fungi.
Data collection	Sequencing data was collected from PacBio sequencing machines at JGI.
Timing and spatial scale	Genomic DNA was extracted from each culture at one timepeoint.
Data exclusions	Several publicly available genomes were excluded from further analyses due to being of too low of quality to compare to other higher quality assemblies. These exclusions are mentioned in the methods sextion.
Reproducibility	Reproducibility was assessed in phylogenetic analyses using bootstrapping methods. Broadly, the results are expected to be reproducible based on detailed descriptions of the methods.
Randomization	Randomization was not performed in this study as it was not relevant to the comparative genomics methods performed in this study. However, relationships between fungal hosts and MRE were not assumed, but were tested using phylogentics.
Blinding	Blinding was not relevant to the study performed here.

Did the study involve field work? ☐ Yes ☒ No

Reporting for specific materials, systems and methods

We require information from authors about some types of materials, experimental systems and methods used in many studies. Here, indicate whether each material, system or method listed is relevant to your study. If you are not sure if a list item applies to your research, read the appropriate section before selecting a response.

Materials & experimental systems

n/a	Involved in the study
☒	☐ Antibodies
☒	☐ Eukaryotic cell lines
☒	☐ Palaeontology and archaeology
☒	☐ Animals and other organisms
☒	☐ Clinical data
☒	☐ Dual use research of concern
☒	☐ Plants

Methods

n/a	Involved in the study
☒	☐ ChIP-seq
☒	☐ Flow cytometry
☒	☐ MRI-based neuroimaging
